# Clinical Significance of IL-23 Regulating IL-17A and/or IL-17F Positive Th17 Cells in Chronic Periodontitis

**DOI:** 10.1155/2014/627959

**Published:** 2014-11-30

**Authors:** Zhenhua Luo, Hui Wang, Yunlong Wu, Zheng Sun, Yafei Wu

**Affiliations:** ^1^Department of Periodontics, School of Stomatology, Capital Medical University, Beijing 100050, China; ^2^State Key Laboratory of Oral Diseases, West China College of Stomatology, Sichuan University, Chengdu 610041, China; ^3^Department of Oral Medicine, School of Stomatology, Capital Medical University, Beijing 100050, China

## Abstract

*Objective*. To investigate the expression level and clinical significance of (IL-17A^+^ and/or IL-17F^+^) Th17 cells under IL-23 regulation in patients of chronic periodontitis (CP) and healthy controls (HC). *Materials and Methods*. The whole peripheral blood samples were collected from 30 CP patients and 25 healthy controls. Flow cytometry was used to test the (IL-17A^+^ and/or IL-17F^+^) Th17 expression level. Recombinant human IL-23 (rhIL-23) was used to detect Th17 differentiation and expansion. Correlation coefficient analysis between Th17 expression level and clinical parameters was analyzed by SPSS software. *Results*. Flow cytometry results showed that IL-17A^+^IL-17F^−^ and IL-17A^−^IL-17F^+^ Th17 were both increased in CP group than in HC group (*P* < 0.01), while, under recombinant human IL-23 (rhIL-23) stimulation, the number of IL-17A^+^IL-17F^−^ Th17 cells was significantly increased in both CP and HC groups (*P* < 0.01). Interestingly, IL-17A^−^IL-17F^+^ Th17 cells were only increased in CP group after rhIL-23 stimulation. Additionally, correlation coefficient analysis showed significant correlation between IL-17A^+^IL-17F^−^ Th17 cell and attachment loss or probing depth (*P* < 0.05). *Conclusions*. This study indicates that both the IL-17A^+^IL-17F^−^ and IL-17A^−^IL-17F^+^ Th17 cells may be involved in pathogenesis of periodontitis. The role of these Th17 cells in the disease pathogenesis needs to be further investigated.

## 1. Introduction

Periodontitis is the most popular oral chronic inflammatory disease, characterized by the destruction of periodontal tissues around the teeth. In chronic periodontitis patients, cytokines produced by immunocytes can regulate the immune responses to distinct periodontal bacteria like actinobacillus actinomycetemcomitans or porphyromanas gingivalis, and further to play the protective or devastating role in disease progression [1, 2]. Though the immune mechanism in periodontitis pathogenesis was still not clear, inflammation mediated by CD4^+^ T lymphocytes was already regarded as an important feature of the pathogenesis [3, 4].

Th17, the novel population of CD4^+^ T cell subsets, has been found more recently for its unique ability to produce interleukin-17 (IL-17, also called IL-17A) [5]. The major role of Th17 cells and their effective cytokine IL-17 has now been described in various models of inflammation mediated destruction and autoimmune diseases, like Crohn's disease or rheumatoid arthritis [6]. Furthermore, previous reports also showed that Th17 cell subset may play a novel important role in pathogenesis of cell-mediated tissue damage caused by immune responses against microbial infection [7]. However, other investigations showed that Th17 cells may produce IL-17A to play protective role by neutrophils recruitment to destructed area in periodontitis lesions [8]. Though study on effect of Th17 cells in periodontitis pathogenesis recently attracted much more attention, the specific role of Th17 cell in periodontitis is still disputed now [9, 10].

IL-17F recently has drawn much more attention due to its great similarity to IL-17A and can be secreted as homodimers or heterodimers with IL-17A [11]. The genes encoding IL-17A and IL-17F are localized in the same chromosomal region and are coexpressed by Th17 cells [12]. Similar to IL-17A, IL-17F can also utilize IL-17RA and IL-17RC receptor complex as its signal transducers to induce the expression of proinflammatory cytokines in many human cell types [13]. IL-23 is a newly discovered cytokine and played great important role in differentiation and proliferation of Th17 cells [14, 15]. Moreover, IL-23 was also found to be involved in human diseases including periodontal diseases. For example, the previous studies demonstrated that both IL-23 mRNA and protein were elevated significantly in periodontal lesions than in control sites [16, 17]. However, we still do not know whether increased cytokine IL-23 in periodontitis would influence the Th17 expansion and differentiation.

In our previous studies of periodontitis pathogenesis, we found that serum IL-17A level was elevated in periodontitis patients than healthy controls [18]. However, to the extent of our knowledge, there are still less reports focused on the correlation between (IL-17A^+^ and/or IL-17F^+^) Th17 cells and periodontitis. So, we did not know whether (IL-17A^+^ and/or IL-17F^+^) Th17 cells may participate in the disease pathogenesis. Therefore, the present clinical study was designed to detect the expression levels of (IL-17A^+^ and/or IL-17F^+^) Th17 cells in periodontitis patients. Furthermore, we would also investigate, under recombinant IL-23 (rhIL-23) stimulation, whether more (IL-17A^+^ and/or IL-17F^+^) Th17 cells would be produced in disease pathogenesis. And are there any significant correlations between expression levels of (IL-17A^+^ and/or IL-17F^+^) Th17 cells and periodontal clinical parameters?

## 2. Subjects and Methods

### 2.1. Study Samples

This study was approved by the Institutional Review Board of West China College of Stomatology in Sichuan University (Protocol number S2011108), with informed consent by participants in this study. Careful periodontal examinations were performed at 6 sites per tooth (mesiobuccal, midbuccal, and distobuccal; mesiolingual, midlingual, and distolingual) to record the parameters including clinical attachment loss (AL), probing depth (PD), Silness-Löe plaque index (PI), and Löe-Silness gingival index (GI) scores. The clinical attachment loss (AL) was measured with the distance from depth of periodontal pocket to the cementoenamel junction. The measurement of probing depth (PD) was determined by measuring distance from a gingival margin to the base of the periodontal pocket with a calibrated periodontal probe. All CP patients were enrolled from our Department of Periodontics, with clinically diagnosed and confirmed as chronic periodontitis disease (with pathological attachment loss and probing depth more than 3 mm) by the periodontists. Demographic characteristics and clinical parameters of both groups were listed in Table 1. After careful examination, the whole blood sample was drawn for the peripheral blood mononuclear cell (PBMC) extraction.

### 2.2. Isolation and Identification of CD4^+^ T Lymphocytes

The peripheral blood mononuclear cell (PBMC) extraction was performed by lymphocytes separation kit (Histopaque 10771, Sigma, USA). Separated PBMC cells from human peripheral blood were labeled with the anti-human CD4^+^ magnetic particles (Cat. number 557767, BD Bioscience, San Diego, CA, USA). Human CD4^+^ T lymphocytes were magnetically separated from PBMC on the BD IMagnet (Cat. number 552311, BD Bioscience) according to the instruction protocol. The purity of CD4^+^ T cells should be more than 95% before next step test. To demonstrate the efficiency of the enrichment and selection steps, cells were stained with APC mouse anti-human CD4 (Cat. number 555349, BD Bioscience) and FITC mouse anti-human CD3 (Cat. number 555332, BD Bioscience). Isotype control was stained by Simultest IgG2a/IgG1 (Cat. number 340394, BD Bioscience). Flow cytometry was performed on a Beckman Coulter FC500 flow cytometry system.

### 2.3. Culture and Stimulation of CD4^+^ T Lymphocytes

CD4^+^ T cell subsets were cultured in RPMI 1640 media containing 10% FBS (Gibco, USA), 50 U/mL penicillin, 50 *μ*g/mL streptomycin, with 1 × 10^6^ cells/mL, in 37°C, 5% CO_2_ condition. CD4^+^ T cells isolated from PBMC were enriched with 2 *μ*g/mL anti-CD3 (Cat. number 555336, BD Bioscience) and 2 *μ*g/mL anti-CD28 (Cat. number 555725, BD Bioscience) monoclonal antibody and 50 ng/mL recombinant human IL-2 (Cat. number 554603, BD Bioscience). CD4^+^ T cells were then stimulated as described above and subsequently subjected to CD4^+^ magnetic separation to increase the purity to >95%. Human recombinant IL-23 (rhIL-23) was added into the supernatant of CD4^+^ T cells with a final concentration of 20 ng/mL to stimulate the CD4^+^ differentiation and expansion for 24 hours. Each sample was divided into two groups according to receiving rhIL-23 stimulation or not.

### 2.4. Flow Cytometry Detection of IL-17A^+^ and/or IL-17F^+^ Th17 Cells

After stimulation with rhIL-23 for 24 hours, cells were cultured in the presence of 50 ng/mL phorbol 12-myristate 13-acetate (P1585, Sigma St. Louis, MO, USA) and 500 ng/mL ionomycin (I0634, Sigma, USA) and with the protein transport inhibitor BD Golgistop 0.7 *μ*L/mL (Cat. number 554714, BD Bioscience) to prevent the cytokine secretion and allow cytokine accumulation inside the cell. Then, CD4^+^ T cells were cultured under condition of 37°C, 5% CO_2,_ to incubate for 4 hours. Cells were fixed and permeabilized with BD Cytofix/Cytoperm Wash Fixation Solution (Cat. number 554715, BD Bioscience) to allow fluorescent antibodies to enter to bind the targeted cytokines. Using BD Cytofix/Cytoperm (Cat. number 554715, BD Bioscience) to perform the cell fixation and wash buffer. Cells were stained with PE Mouse anti-Human IL-17A (Cat. number 560436, BD Bioscience) and Alexa Fluor 488 Mouse anti-Human IL-17F (Cat. number 561331, BD Bioscience). Isotype control was stained with Simultest IgG2a/IgG1 (Cat. number 340394, BD Bioscience). Flow cytometry was performed on a Beckman Coulter FC500 flow cytometry system.

### 2.5. Statistical Analysis

All data were analyzed by Statistical Package for Social Sciences version 14.0 software (SPSS Inc., Chicago, IL, USA). The chi-square test was used to compare differences in gender in Table 1. Student's* t*-test was used to compare the means of two samples with both groups. Correlation coefficients were carried out with Spearman's correlation coefficient analysis, where *P* < 0.05 was considered to be statistically significant.

## 3. Results

### 3.1. Expression Level of IL-17A^+^IL-17F^−^ and/or IL-17A^−^IL-17F^+^ Th17 Cells in CP and HC Groups

**Figure 1:**
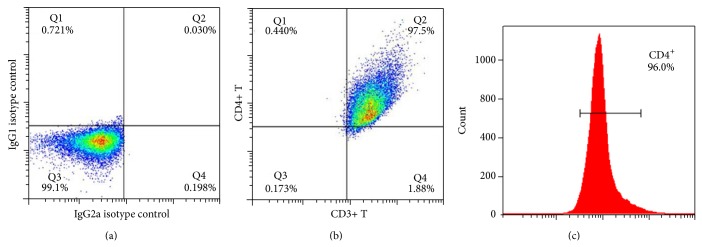
Purity verification of separated CD4^+^ T cells by flow cytometry. (a) showed the isotype control performed by Simultest IgG2a/IgG1. (b) represents double staining by anti-human CD3 and CD4 antibody. (c) represents anti-human antibody CD4 staining. These results showed that the purity of CD4^+^ from PBMC can reach a percent of more than 95%.

**Figure 2:**
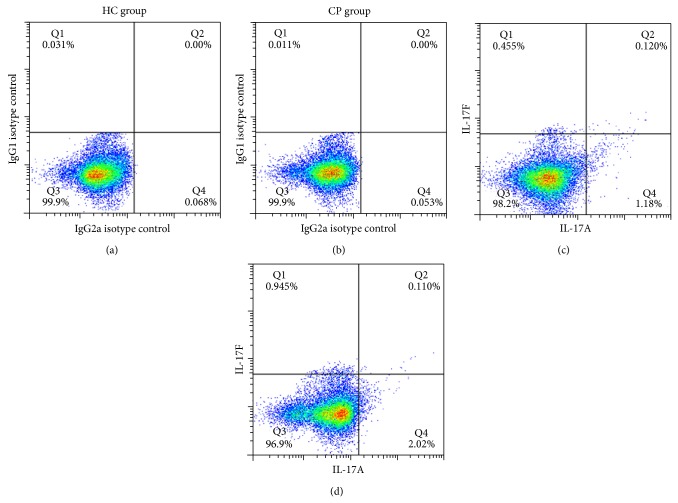
Representative dot blot showing percent expression level of (IL-17A^+^ and/or IL-17F^+^) Th17 cells in CP group (*n* = 30) and HC group (*n* = 25) within the CD4^+^ population. (a) and (b) showed the isotype control performed by Simultest IgG2a/IgG1, while (c) and (d) showed the double staining performed by IL-17A and IL-17F anti-human antibody.

**Figure 3:**
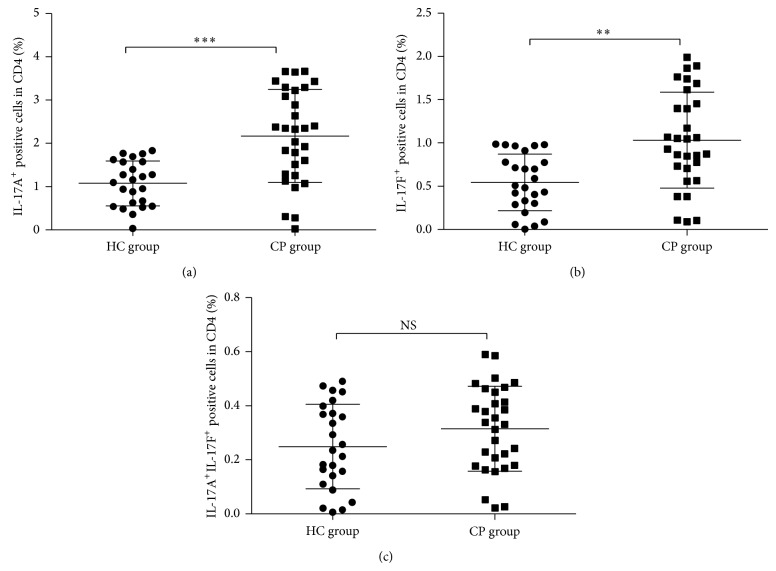
Statistical analysis of IL-17A^+^ and/or IL-17F^+^ Th17 cells expression level in CP and HC groups. ((a) IL-17A^+^IL-17F^−^ Th17 cell; (b) IL-17A^−^IL-17F^+^ Th17 cell; and (c) IL-17A^+^IL-17F^+^ Th17 cell.)

Separated CD4^+^ T cells were validated by anti-human CD3 and CD4 flow cytometry antibody to test CD4 cell purity. The results showed that the purity of CD4^+^ T from PBMC can reach a percent of more than 95% (Figure 1). Flow cytometry of the sample staining with IL-17A and IL-17F antibody was demonstrated in Figure 2 and further data analysis was shown in Figure 3. Flow cytometry analysis showed significantly elevated levels of IL-17A^+^IL-17F^−^ Th17 cell (Figure 3(a), *P* < 0.01) and IL-17A^−^IL-17F^+^ Th17 cell in CP group compared with HC group (Figure 3(a), *P* < 0.01). However, it did not demonstrate statistically significant difference between CP group and HC group with the IL-17A^+^IL-17F^+^ double positive Th17 cell (Figure 3(a), *P* > 0.05).

### 3.2. Expression Level of IL-17A^+^IL-17F^−^ and/or IL-17A^−^IL-17F^+^ Th17 cells in CP and HC Groups under rhIL-23 Stimulation

**Figure 4:**
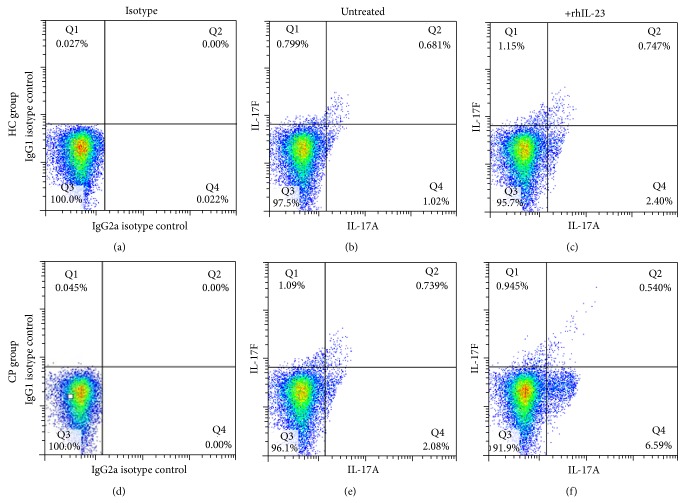
Expression level of (IL-17A^+^ and/or IL-17F^+^) Th17 cells in CP and HC groups with or without recombinant IL-23 stimulation. (a) and (d) showed the isotype control performed by Simultest IgG2a/IgG1, while (b), (c), (d), and (e) showed the double staining performed by IL-17A and IL-17F anti-human antibody ((c) and (f) groups were treated by rhIL-23, while (b) and (e) were not). HC group includes (a), (b), and (c); CP group includes (d), (e), and (f).

**Figure 5:**
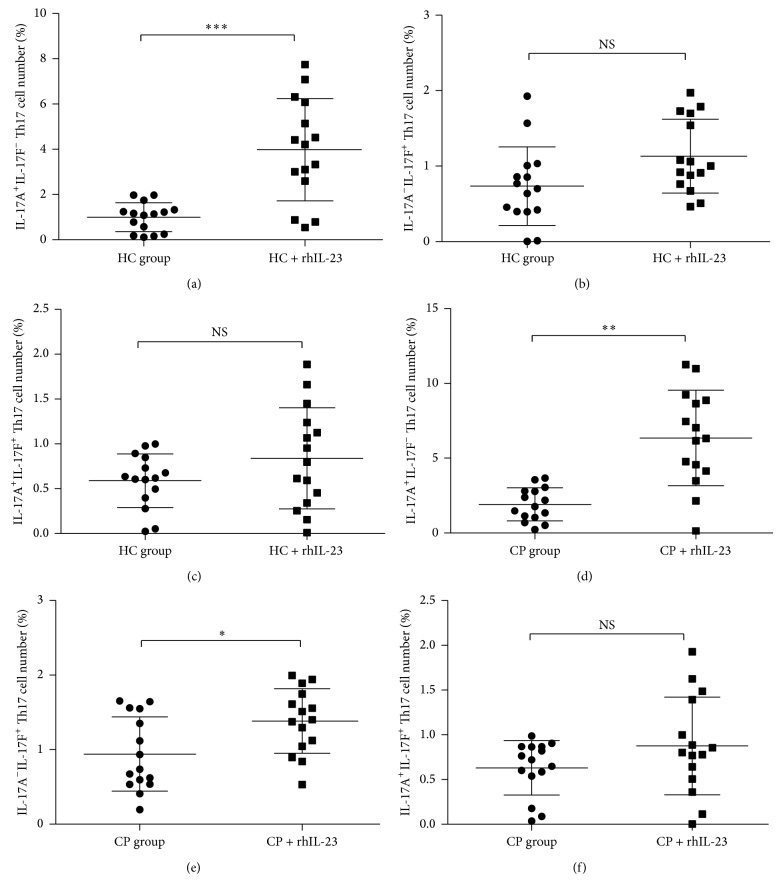
Statistical analysis of IL-17A^+^ and IL-17F^+^ Th17 cells expression in CP (*n* = 15) and HC groups (*n* = 15) with or without recombinant IL-23 stimulation. ((a) and (d) IL-17A^+^IL-17F^−^ Th17 cell; (b) and (e) IL-17A^−^IL-17F^+^ Th17 cell; and (c) and (f) IL-17A^+^IL-17F^+^ Th17 cell.)

CD4^+^ T cells were separated from CP patients and HC group peripheral blood with CD4^+^ magnetic separation kit to increase the purity and were cultured under rhIL-23 stimulation for 24 hours in vitro. Flow cytometry analysis was used to test the percent change in several Th17 cells (Figure 4). It showed a significantly elevated level of IL-17A^+^IL-17F^−^ Th17 cell (Figure 5(a), *P* < 0.01) in CP group and HC group after rhIL-23 stimulation. However, there is only IL-17A^−^IL-17F^+^ Th17 cell number significantly increased in CP group after rhIL-23 simulation (Figure 5(b), *P* < 0.05), not in HC group (Figure 5(c), *P* > 0.05). Considering IL-17A^+^ IL-17F^+^ Th17 cell number change after rhIL-23 stimulation, no statistical significance was to be found in CP group and HC group after rhIL-23 stimulation (Figure 5(c), *P* > 0.05).

**Figure 6:**
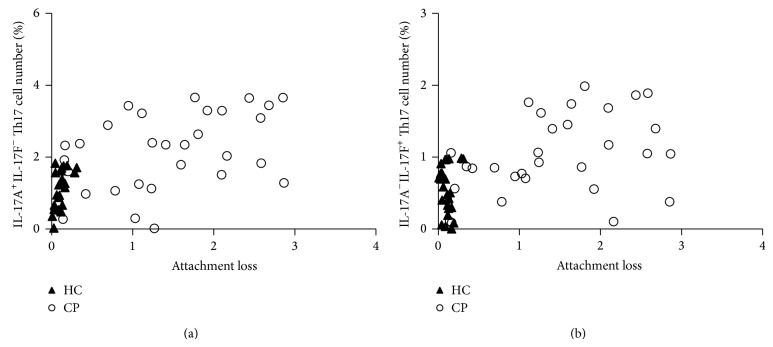
Plot of IL-17A^+^IL-17F^−^ and IL-17A^−^IL-17F^+^ Th17 cells number as a function of attachment loss in chronic periodontitis. (a) represented IL-17A^+^IL-17F^−^Th17 cell and (b) represented IL-17A^−^IL-17F^+^ Th17 cell. The open circles denoted CP patients (*n* = 30), while closed triangles denoted periodontally healthy control individuals (*n* = 25).

IL-17A^+^IL-17F^−^ and IL-17A^−^IL-17F^+^ Th17 cell number were plotted as a function of attachment loss for the CP patients (Figure 6). It showed obviously that CP patients demonstrated increased IL-17A^+^IL-17F^−^ and IL-17A^−^IL-17F^+^ Th17 cells compared with levels in healthy individuals.

### 3.3. Correlation Coefficients of IL-17A^+^IL-17F^−^ and/or IL-17A^−^IL-17F^+^ Th17 Cells Expression Level with Clinical Parameters

The correlation coefficient analysis showed positively significant correlations of IL-17A^+^IL-17F^−^ Th17 cells with attachment loss and probing depth (Table 2, *P* < 0.05). It also showed a significant correlation between IL-17A^−^IL-17F^+^ Th17 cells and attachment loss in CP group (Table 2, *P* < 0.05). However, there was no significant correlation of IL-17A^−^IL-17F^+^ Th17 cell number and probing depth of CP patients (Table 2, *P* > 0.05). Additionally, it also showed no significant correlation of two Th17 cells and plaque index or gingival index (Table 2, *P* > 0.05).

## 4. Discussion

In recent years, Th17 cell has attracted more and more attention of researchers and was identified to be present in peripheral blood and local lesions of periodontitis patients [19, 20]. Though our previous studies have focused on the expression level of Th17 associated cytokines in periodontitis patients, we still do not know the expression level of IL-17A^+^IL-17F^−^ and/or IL-17A^−^IL-17F^+^ Th17 cells in CP patients compared to healthy controls. Therefore, this is the first study reporting elevated levels of IL-17A^+^IL-17F^−^ and IL-17A^−^IL-17F^+^ Th17 cells in chronic periodontitis. The present results have laid the basis to understand potential role of IL-17A^−^IL-17F^+^ Th17 in the pathogenesis of periodontitis.

Previous in vitro test supports the hypothesis that Th1 cell was associated with periodontal stable lesions, while Th2 cells with disease progression [21]. For example, previous reports showed that a destructive role for distinct Th cells throughout periodontitis refers to the Th2 subset, which also presented in periodontal lesions [22]. However, besides Th1 and Th2 cells, recent investigations also demonstrated that the Th17 subset may play osteoclastogenic effect [23]. Th17 cells developed through cytokine signals distinct from and antagonized by products from Th1 and Th2 subsets [24, 25]. Reports showed that Th17 was involved in periodontal bone destruction via IL-17 production [26] and might be suppressed by molecular De-1 (developmental endothelial locus 1) [27], indicating the emerging role in periodontitis pathogenesis.

In vitro test showed that human recombinant IL-23 can significantly enhance the expression level of IL-17A^+^IL-17F^−^ and IL-17A^−^IL-17F^+^ Th17 cells both in CP group and in HC group. However, to extent of our knowledge, precise role of IL-23 in local periodontal lesions is still obscure. In our previous study, we found the increased levels of IL-23 expression in diseased periodontal lesions, which suggested its involvement in the pathogenesis of periodontal destruction [17, 28]. Previous reports showed that Th17 cells expressed IL-17 in an IL-23-dependent fashion [14]. Additionally, in our previous study, we did find significant elevated IL-23 expression level in local lesions in CP patients [18]. However, though serum IL-23 was not elevated significantly in CP, we still found an increased level of serum IL-17 in CP patients. This may be attributed to other Th17 promoting cytokines like IL-1 or IL-6 [14].

As previously reported, the periodontal diseases pathogenesis may not simply be accompanied by a consequence of Th1/Th2 balance, but these profiles of immune response may also be profoundly influenced by other subsets like Th17, which may play indispensable role in periodontitis. Together with conclusions of our previous studies, the present results have reconfirmed that the expression patterns of Th17 and its cytokines may be related to the clinical parameters of periodontitis and may be involved in the disease progression. As indicated above, chronic periodontitis demonstrated a chronic inflammation involved in the hazardous microbes infection and the consequent immune-inflammatory responses. Therefore, in the present study, we described the presence of three distinct Th17 cells in CP and HC groups. Apparently, the increased levels of IL-17A^+^IL-17F^−^ and IL-17A^−^IL-17F^+^ Th17 cells in CP group correlated with attachment loss and probing depth, indicating there may be potential mechanism for these Th17 cells in periodontitis.

IL-17F recently has drawn much more attention due to its great similarity to IL-17A, and the genes encoding IL-17A and IL-17F are localized in the same chromosomal region and are coexpressed by Th17 cells [11, 12]. Similar to IL-17A, IL-17F can also utilize IL-17RA and IL-17RC receptor complex as its signal transducers to induce the expression of proinflammatory cytokines in many human cell types [13]. In our present study, the flow cytometry results showed that there were significantly elevated levels of IL-17A^+^IL-17F^−^ and IL-17A^−^IL-17F^+^ Th17 cells in CP group compared to HC group. Therefore, IL-17A^−^IL-17F^+^ Th17 cells may also play important role in periodontal disease like IL-17A^+^IL-17F^−^ Th17 cell as both Th17 cells were correlated with the attachment loss in periodontitis. When under rhIL-23 stimulation, more IL-17A^+^IL-17F^−^ Th17 cells would be produced in CP group than in HC group, and also only in CP group the change of IL-17A^−^IL-17F^+^ Th17 showed a significantly increase than in HC group. Therefore, the present study indicated to us that both IL-17A^+^IL-17F^−^ and IL-17A^−^IL-17F^+^ Th17 cells were totally involved in disease pathogenesis of CP group.

Taken together, this clinical study indicated that IL-17A^+^ and/or IL-17F^+^ positive Th17 may participate in the periodontitis pathogenesis. Further epidemiological study between Th17 cells and periodontal diseases should be investigated, and possible pathological relationships should be further studied. Meanwhile, in further study, we should pay more attention to the role of Th17 associated cytokines IL-17A and IL-17F to test how these two cytokines affect the periodontal ligament fibroblast, through which signaling pathways the cytokine IL-17A and IL-17F may affect the pro-inflammatory mediator release.

## Figures and Tables

**Table 1:** Demographic characteristics and clinical parameters in HC and CP groups.

	Age^a^ (years)	Gender^b^		PD^a^ (mm)	AL^a^ (mm)	PI^a^	GI^a^
		Male	Female				
HC group (mean ± SD)	38.5 ± 10.2	12 (48%)	13 (52%)	1.7 ± 0.9	0.1 ± 0.1	0.2 ± 0.3	0.5 ± 0.4
CP group (mean ± SD)	42.4 ± 11.0	15 (50%)	15 (50%)	4.5 ± 1.5	1.4 ± 0.9	1.1 ± 0.5	1.2 ± 0.6
*P* value	NS	NS	NS	*P*< 0.001	*P*< 0.001	*P*< 0.001	*P*< 0.001

^a^Student's *t*-test; ^b^chi-square test.

HC group: healthy controls; CP group: chronic periodontitis; SD: standard deviation; PD: probing depth; AL: attachment loss; PI: plaque index; GI: gingival index; NS represents no statistically significant difference (*P* > 0.05).

**Table 2:** Correlation coefficients (*R* squared) between two distinct Th17 cells (IL-17A^+^IL-17F^−^ and IL-17A^−^IL-17F^+^ Th17) expression level and clinical parameters of CP and HC groups.

Groups	IL-17A^+^IL-17F^−^ Th17		IL-17A^−^IL-17F^+^ Th17	
	HC	CP	HC	CP
Probing depth (PD)	0.03	0.21^*^	0.04	0.01
Attachment loss (AL)	0.08	0.17^*^	0.009	0.15^*^
Plaque index (PI)	0.06	0.05	0.09	0.08
Gingival index (GI)	0.10	0.02	0.03	0.11

^*^Correlation is significant at *P* < 0.05 level (Spearman correlation coefficient).
